# Persistence of *Salmonella* Typhimurium LT2 in Soil Enhanced after Growth in Lettuce Medium

**DOI:** 10.3389/fmicb.2017.00757

**Published:** 2017-04-28

**Authors:** Eva Fornefeld, Jasper Schierstaedt, Sven Jechalke, Rita Grosch, Adam Schikora, Kornelia Smalla

**Affiliations:** ^1^Julius Kühn-Institut, Federal Research Centre for Cultivated Plants, Institute for Epidemiology and Pathogen DiagnosticsBraunschweig, Germany; ^2^Leibniz Institute of Vegetable and Ornamental CropsGroßbeeren, Germany; ^3^Institute of Phytopathology, Justus-Liebig University GiessenGiessen, Germany

**Keywords:** preadaptation, lettuce medium, survival, *Salmonella enterica*, soil, biofilm, qPCR

## Abstract

The persistence of *Salmonella* in the environment is influenced by a multitude of biotic and abiotic factors. In addition, its persistence can be influenced by preadaptation before the introduction into the environment. In order to study how preadaptation changes the survival of *Salmonella* in soil and therefore its potential to colonize the phytosphere, we developed a new medium based on lettuce material [lettuce medium (LM)]. *Salmonella enterica* serovar Typhimurium strain LT2 was used as a model for *Salmonella* in this study. LT2 was inoculated into soil microcosms after pregrowth in Luria Bertani (LB) broth or in LM. Survival of LT2 in soil was monitored over 56 days by plate counts and quantification of the Typhimurium-specific gene STM4497 using qPCR in total community DNA for which primers and TaqMan probe were designed in this study. Significantly enhanced persistence was observed for LT2 pregrown in LM compared to LT2 pregrown in LB, indicating a preadaptation effect. Surprisingly, no improved survival could be observed for *S.* Typhimurium strain 14028s and *S. enterica* serovar Senftenberg after pregrowth on LM. This indicates a high strain specificity of preadaptation. Results from previous studies suggested that biofilm formation could enhance the survival of human pathogens in various environments and might contribute to enhanced survival on plants. *In vitro* biofilm assays with several *Salmonella* strains revealed a strain-specific effect of LM on the biofilm formation. While LM significantly improved the biofilm formation of *S.* Senftenberg, the biofilm formation of LT2 was better in LB. This indicates that the better survival of LM-pregrown LT2 in soil was not linked to an improved ability to form biofilms but was likely due to other factors. Most importantly, this study showed that the medium used to pregrow *Salmonella* can influence its survival in soil and its biofilm formation which might influence the fate of *Salmonella* in soil.

## Introduction

Bacterial human pathogens (HPs) on fresh produce are a potential threat to human health. *Salmonella enterica* is among the most frequent bacterial HP and can cause life-threatening diarrheal diseases. As specific decontamination of *S. enterica* from fruits and vegetables is practically impossible, contamination should be avoided or reduced. Unfortunately, contaminations of produce with *S. enterica* can occur along the whole production chain and one possible source is the soil (Guo et al., 2002; Klerks et al., 2007; Barak et al., 2011; Kroupitski et al., 2011; Olaimat and Holley, 2012). Occurrence of *S. enterica* in soil can originate from irrigation or flooding water, organic fertilizers, feces or plant residues plowed into soil after harvest (Semenov et al., 2010; Jacobsen and Bech, 2012; Olaimat and Holley, 2012). Once introduced into the soil, *S. enterica* might be able to persist for months (Danyluk et al., 2008; Brennan et al., 2014). Its survival in soil is influenced by a multitude of biotic and abiotic factors. General statements about the impact of a single factor are difficult, since different studies often led to contradictory findings, probably due to complex interactions between influencing factors, differences in experimental setups, strains or detection and quantification methods. Among others, preadaptation prior to their introduction into the soil environment might be important for the fate of *S. enterica* in soil. For example, Semenov et al. (2010) observed an improved survival of *S. enterica* serovar Typhimurium (*S*. Typhimurium) in dung after cells had passed the digestive tract of cows. Similarly, after exposure to sublethal acidic conditions, *S.* Typhimurium showed improved survival in subsequent extremely acidic conditions (Lee et al., 1995). Furthermore, Shah et al. (2013) found that preadaptation of *S.* Typhimurium to cold resulted in an increased survival in acidic conditions. Preadaptation to peanut oil or exposure to short sublethal heat stress was found to increase heat resistance of *S. enterica* serotypes Typhimurium, Enteritidis, Tennessee, Thompson, and Hartford (Fong and Wang, 2016b), and Leyer and Johnson (1992) observed enhanced survival of *S.* Typhimurium strain LT2 in cheese after adaptation to acid stress. The preadaptation effect is also termed cross-protection, a term that is commonly used in studies related to the food industry. As reviewed by Andino and Hanning (2015), exposure to desiccation stress can lead to cross-tolerance of *S. enterica* serotypes Typhimurium, Enteritidis, Newport and Infantis to other stresses like heat disinfectants and UV light.

Although these results suggest an important role of preadaptation for the survival of bacterial HP, in the majority of published studies not much attention is paid to the conditions under which the inoculum is grown. Often the inoculum is pregrown in a rich medium such as the Luria Bertani (LB) broth.

The aim of this study was to analyze whether pregrowth of the model strain *S. enterica* serovar Typhimurium strain LT2 in a newly developed lettuce medium (LM) improves its survival in soil compared to pregrowth in LB. The LM was developed because our long-term goal is to understand the factors influencing the ability of *Salmonella* to colonize the phytosphere. Furthermore, a medium containing plant material simulates incubation of *Salmonella* in plant residues that remain on the field after harvest and are plowed into the soil before the next crop is planted. Survival of LT2 pregrown in LM or LB was studied in soil microcosms. The fate of LT2 was monitored by plate counts and qPCR. In an additional experiment, soil survival of LT2, *S*. Typhimurium strain 14028s and *S. enterica* serovar Senftenberg pregrown either in LM or LB was followed only by plate counts. Knowing that biofilm formation resulted in increased survival to desiccation and gastric stresses (Aviles et al., 2013) and increased persistence in food production environments (Vestby et al., 2009) it was suggested that biofilm formation can enhance survival of HP on plants (Yaron and Römling, 2014), we hypothesized, that biofilm formation might influence the survival of *Salmonella* in soil. To test if LM can influence the biofilm formation ability a set of *S. enterica* serovars was analyzed using *in vitro* biofilm assays on cells grown in LM or LB broth.

## Materials and Methods

### Bacterial Strains

*Salmonella enterica* serovar Typhimurium LT2 DSM 18522 was obtained from the DSMZ (Braunschweig, Germany) and *S. enterica* serovar Typhimurium 14028s was obtained from Dr. Isabelle Virlogeux-Payant (INRA Tours, France). *S. enterica* serovars Senftenberg, Brunei, and Muenster (Elviss et al., 2009) were kindly provided by Dr. Nicola Holden (The James Hutton Institute, Dundee, Scotland, UK) and Prof. John Coia (Scottish Reference Centre for Salmonella, Glasgow, Scotland, UK). Spontaneously rifampicin-resistant mutants were isolated by inoculation of an overnight culture on LB agar plates containing rifampicin (50 mg/L). Resistant colonies were plated onto LB agar with rifampicin to confirm resistance.

### Lettuce Medium

To prepare sterile lettuce extract, approximately 500 g of lettuce leaves (*Lactuca sativa* L. cultivar Tizian) were shredded with 150 mL dH_2_O in a blender until the plant material was homogenized. This homogenate was filtered through filter paper (MN 615, Macherey-Nagel GmbH & Co. KG, Düren, Germany) to remove larger particles, and centrifuged at 10,000 *g* for 30 min at 4°C. The supernatant was sterile-filtered through 0.22 μm membrane filters (Sterivex, Merck KGaA, Darmstadt, Germany). The LM consists of 25% (v/v) of fresh lettuce extract, 20% M9 salts (Sigma-Aldrich Chemie GmbH, München, Germany; 5x concentrated to obtain the final concentration indicated by the manufacturer) and 55% sterile deionized water. To prepare LM plates, 5% sterile deionized water was added instead of 55% and 50% 2x concentrated water agar.

### Microcosm Experiments

The first microcosm experiment was performed in order to analyze the survival of *S.* Typhimurium LT2 in soil. A sandy soil from Großbeeren (52° 33′ N, 13° 22′ E, Germany), was used. It was characterized as Arenic-Luvisol (Rühlmann and Ruppel, 2005), with less silty sand and 5.5% clay [diluvial sand (DS)] which was sieved (2 mm) and adjusted to 50% of its maximal water holding capacity resulting in a final water content of 112 mL per kg of soil (Schreiter et al., 2014).

LT2 was grown at 37°C overnight in LB broth (Carl Roth GmbH + Co. KG, Karlsruhe, Germany) supplemented with rifampicin (50 mg/L). 250 μL of this culture were transferred to 250 mL of LM or LB, respectively. Cultures were grown overnight for 18–20 h at 37°C. Stationary phase cells were pelleted at low speed to avoid stressing the cells (1,500 *g*, 10 min) and washed twice with 0.85% (w/v) NaCl solution. Soil inoculation was performed by mixing the DS soil with bacterial solutions to obtain a final concentration of 10^6^ colony forming unit (CFU) per g dry soil. Inoculated soil was filled into polystyrene flowerpots (11 cm diameter, 9 cm height) with 600 g soil per pot, and pots were covered to reduce evaporation of water.

The experiment was performed with three different treatments: (i) DS soil with LT2 pregrown in LB with rifampicin (50 mg/L), (ii) DS soil with LT2 pregrown in LM with rifampicin (50 mg/L) and (iii) DS soil without inoculum as control. Each treatment was represented by four replicates. The microcosms were incubated for 35 days at 20°C, and the water content was kept constant at 50% maximal water holding capacity by monitoring the weight and adding water when necessary. Soil was sampled 0, 4, 7, 10, 14, 21, 28, and 35 days post inoculation (dpi). Before sampling the soil in each pot was mixed thoroughly.

The second microcosm experiment was performed in order to compare the survival of LT2 in soil to survival of *S.* Typhimurium strain 14028s and *S. enterica* serovar Senftenberg. In this experiment, each strain was pregrown on LM and LB agar plates supplemented with rifampicin (50 mg/L). The plates were incubated overnight for 18–20 h at 28°C. Bacterial cells were pelleted (1,500 *g*, 10 min) and washed with 10 mM MgCl_2_ solution. Soil inoculation was performed by mixing the DS soil with bacterial solutions to obtain a final concentration of 10^6^ CFU per g dry soil. Inoculated soil was filled into 50 mL conical tubes with 50 g per tube and the tubes were closed to prevent evaporation of water. The experiment was performed with three different treatments for each *Salmonella* strain: (i) DS soil with *Salmonella* pregrown on LB with rifampicin (50 mg/L), (ii) DS soil with *Salmonella* pregrown on LM with rifampicin (50 mg/L) and (iii) DS soil without inoculum as control. Each treatment was represented by four replicates. The microcosms were incubated for 42 days at 20°C. Soil was sampled 0, 4, 7, 10, 14, 21, 28, 35, and 42 dpi. Before sampling the soil in each tube was mixed thoroughly. The experiment was performed in three independent repetitions.

### Quantification of *Salmonella* by Plate Counts and Quantification of *S.* Typhimurium LT2 by Quantitative Real-Time PCR (qPCR)

To count the CFU of LT2 in soil (microcosm experiment), triplicates of serial dilutions of soil samples were dropped on R2A agar (Merck KGaA, Darmstadt, Germany) supplemented with rifampicin (50 mg/L) and cycloheximide (100 mg/L) in the first microcosm experiment, or XLD agar (Carl Roth GmbH + Co. KG, Karlsruhe, Germany) with rifampicin (50 mg/L) in the second microcosm experiment, and colonies were enumerated after incubation at 37°C for 24 h.

In the first microcosm experiment, total community DNA was extracted from 0.5 g soil of the soil samples taken, using the FastDNA SPIN Kit for Soil (MP Biomedicals, Heidelberg, Germany) and purified with GENECLEAN SPIN Kit (MP Biomedicals, Heidelberg, Germany) according to the manufacturer’s instructions. For quantification of *S.* Typhimurium LT2 in total community DNA, the gene STM4497, specific for *S. enterica* Typhimurium, was quantified using qPCR. The qPCR-based quantification was carried out 0, 7, 14, 21, and 28 dpi in the first repetition of the experiment. *S.* Typhimurium LT2 was quantified by qPCR assays using a fragment from LT2 cloned into the pGEM-T vector (Promega, Mannheim, Germany), dilution series as standard. *S.* Typhimurium target DNA was amplified in 50 μL reaction mixture containing 5 μL of 1:5 diluted DNA extracted from the sample, 1.25 U TrueStart Taq DNA polymerase (Fermentas, St. Leon-Rot, Germany), TrueStart buffer, 0.2 mM of each deoxynucleoside triphosphate, 2.5 mM MgCl_2_, 0.1 mg/mL bovine serum albumin (Fermentas), 5% DMSO and 0.3 μM of forward primer LT2-F (GTCAAATAACCCACGTTCA), reverse primer LT2-R (TCTCAAAAACAACGGCTC) and TaqMan probe LT2-PR (FAM-TCGCGCACCTCAACATCT-TAMRA). *S.* Typhimurium-specific primers amplifying a 187 bp fragment of the gene STM4497, which is specific for *S.* Typhimurium (Kim et al., 2006) were developed in this study using the CLC Main Workbench 7 (CLC bio, Aarhus, Denmark). Reactions were run for 10 min at 95°C and 40 cycles of 15 s at 95°C, 15 s at 58.3°C and 60 s at 60°C in the CFX96 System (Bio-Rad, München, Germany).

To compare the survival in soil of *Salmonella* pregrown in LM or LB, slopes of the linear regressions of log transformed CFU and qPCR results were calculated for each replicate. Results were compared by Student’s *t*-test and differences considered significant when *p*-values were lower than 0.05 (Supplementary Table S1).

### *In Vitro* Biofilm Assay

The biofilm assay was carried out as described by Stepanovic et al. (2007). For cell preparation, 5 mL LB broth were inoculated with 3–4 colonies and incubated for 18 h at 37°C. After incubation, the stationary-phase culture was vortexed carefully to break up cell clusters and adjusted to OD_600__nm_ = 1 with LB broth which corresponds to about 10^8^ CFU per mL. This inoculum was diluted 1:100 in LM or LB broth for biofilm cultivation. 96-well microtiter plates containing 200 μL of the inoculated medium for biofilm cultivation (LB or LM, inoculated at a final concentration of 10^6^ CFU/mL) were incubated aerobically at 37°C for 24 h. After washing and fixation, the cells were stained with 0.41% crystal violet. The excess dye was removed by washing with tap water, the dye bound to the cells was dissolved in 95% ethanol and the OD_570__nm_ measured. The mean OD value of the negative controls was subtracted from all sample values. The assay was repeated four times, with eight replicates per strain and medium tested. OD values of growth in LB or LM were compared for each strain using Student’s *t*-test.

## Results

### Pregrowth in Lettuce Medium (LM) Enhanced Survival of *S.* Typhimurium LT2 in Soil

**FIGURE 1:**
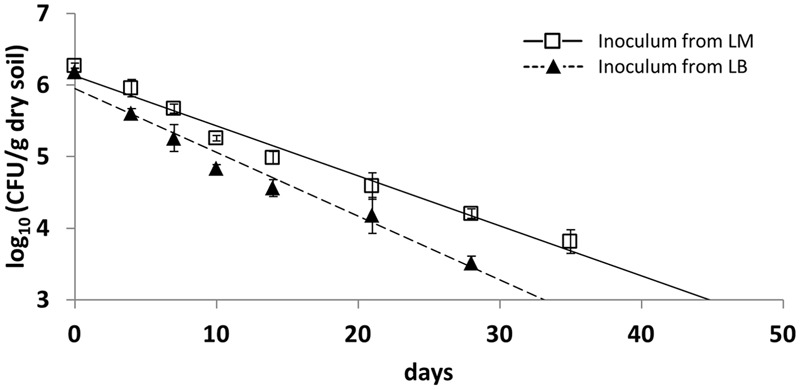
**Colony forming unit (CFU) counts in diluvial sand (DS) per gram soil (dry weight) of *Salmonella enterica* serovar Typhimurium strain LT2 pregrown in Luria Bertani (LB) broth or lettuce medium (LM).** Soil was sampled 0, 4, 7, 10, 14, 21, 28, and 35 days post inoculation (dpi). Lines correspond to linear regressions representing the data to visualize differences observed between pregrowth in LM and LB. Error bars indicate standard deviations of four replicates. The slopes and coefficients of determination of the linear regressions are given in Supplementary Table S1.

Monitoring the survival of LT2 in the first soil microcosm experiment revealed that the CFU number of cultivable LT2 cells pregrown in LB decreased faster than for those pregrown in LM. CFU counts of LT2 were quantifiable on selective plates until 28 and 35 dpi for inoculum from LB and LM, respectively (**Figure 1**). During this time the abundance of cultivable LT2 decreased from the initial 10^7^ CFU/g dry soil to about 10^4^ CFU/g dry soil after 4 weeks for cells pregrown in LB and after 5 weeks for cells pregrown in LM. LT2 pregrown in LM persisted at a significantly higher level in soil compared to LT2 pregrown in LB. CFU counts of cells from LM decreased significantly (*p <* 0.05; Supplementary Table S1) slower if compared to LT2 from LB.

**FIGURE 2:**
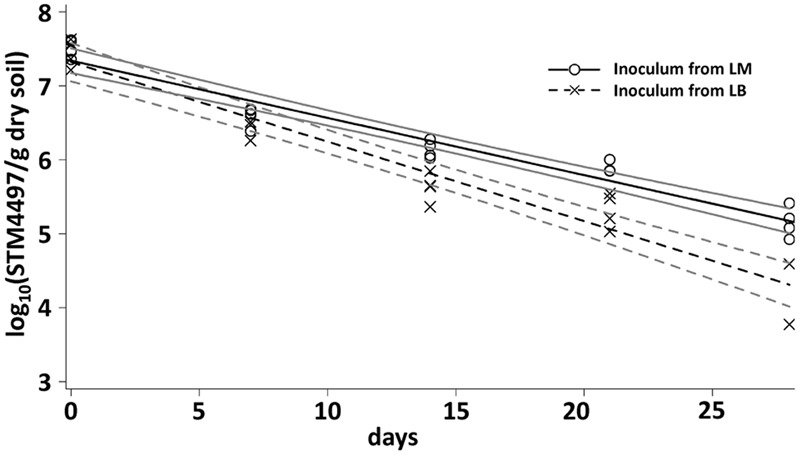
**Quantification of the gene STM4497 specific for *S. enterica* serovar Typhimurium using qPCR was carried out 0, 7, 14, 21, and 28 dpi.** Lines correspond to linear regressions representing the data to visualize differences observed between LT2 pregrown in LM and LT2 pregrown in LB. The slopes and coefficients of determination of the linear regressions are given in Supplementary Table S1. 95% confidence intervals are indicated by gray lines.

The results of the CFU counts were confirmed by a cultivation-independent qPCR-based analysis (**Figure 2**). LT2 cells in soil were quantifiable by qPCR until 28 dpi. The copy number of the target gene decreased from about 10^7^ gene copies per gram of dry soil at the beginning of the experiment to about 10^4^ gene copies per gram of dry soil after 4 weeks. Gene copy numbers in the treatment with LT2 from LM decreased significantly (*p* < 0.05; Supplementary Table S1) slower if compared to the treatment with LT2 from LB.

**FIGURE 3:**
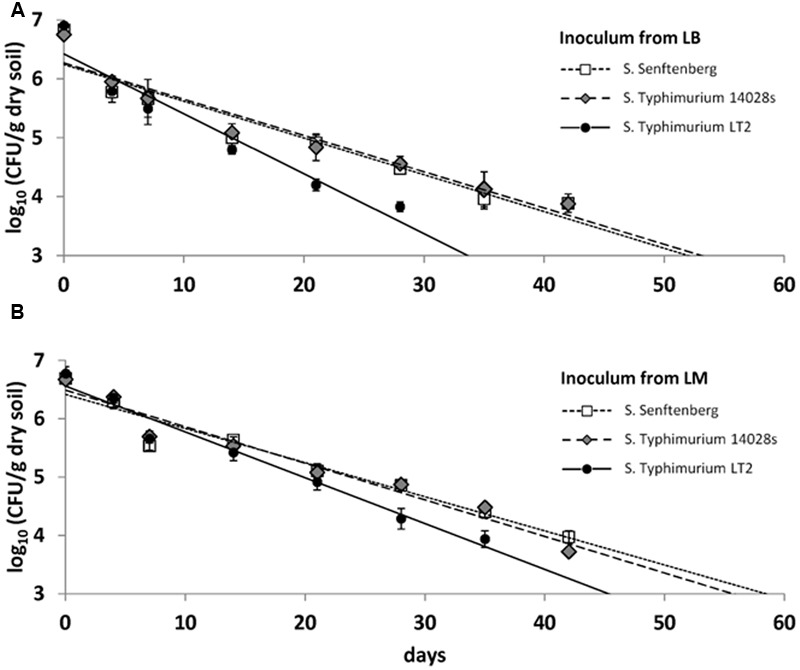
**Colony forming unit counts in DS per gram soil (dry weight) of *S. enterica* serovar Typhimurium strains LT2 and 14028s as well as *S. enterica* serovar Senftenberg inoculated from Luria Bertani (LB) broth (A)** and LM **(B)**. Soil was sampled 0, 4, 7, 10, 14, 21, 28, 35, 42, 49, and 56 dpi. Lines correspond to linear regressions representing the data to visualize differences observed for the strains. Error bars indicate standard deviations of four replicates. The slopes and coefficients of determination of the linear regressions are given in Supplementary Table S1.

In the second soil microcosm experiment, survival of LT2 was compared to survival of *S*. Typhimurium strain 14028s and *S*. Senftenberg, each pregrown on LM or on LB. CFU counts were quantifiable until 42 dpi and they decreased from the initial 10^7^ CFU/g dry soil to about 10^4^ CFU/g dry soil after 6 weeks (**Figure 3**). The results from the first microcosm experiment were confirmed for LT2 in the second microcosm experiment as LT2 pregrown in LM persisted in soil again at significantly higher numbers compared to LT2 pregrown in LB. For *S*. Typhimurium 14028s and *S*. Senftenberg, no significant differences between inoculum from LB and LM were found (Supplementary Table S1). The soil microcosm experiment was carried out three times, confirming the significantly lower survival rate of LT2 from LB compared to LT2 from LM in the repetitions as well as results for *S*. Typhimurium 14028s and *S*. Senftenberg (Supplementary Figure S1).

### Growth in LM Affects the Biofilm Formation

**FIGURE 4:**
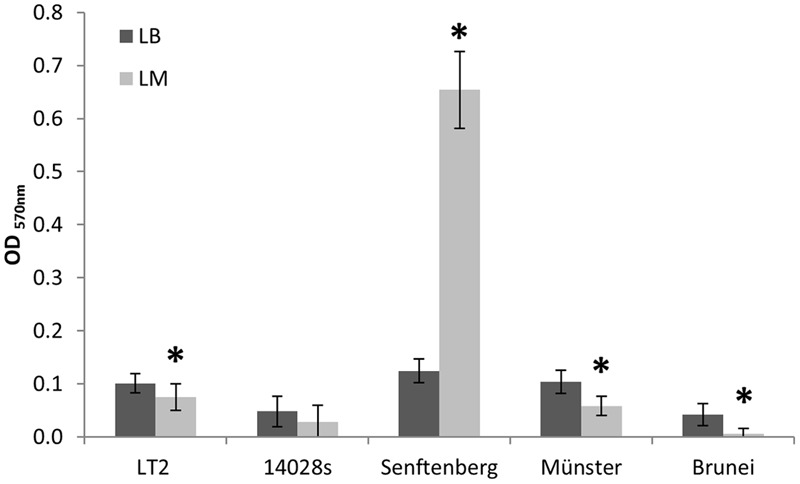
***In vitro* biofilm assay of *S. enterica* serovar Typhimurium strains LT2, 14028s, and *S. enterica* serovars Senftenberg, Münster, and Brunei.** OD_570__nm_ values corresponding to biofilm formation in Luria Bertani (LB) broth are indicated as dark bars and values for LM as light bars. The mean OD value of the negative controls was subtracted from all sample values. Error bars indicate standard deviations of 32 replicates (four independent repetitions of the assay with eight replicates each). Significant differences between treatments for each strain are indicated by asterisk (Student’s *t*-test, *p <* 0.05).

In order to test the hypothesis that growth in LM affects the ability of *S. enterica* strains to form biofilms, we analyzed biofilm formation of LT2, *S.* Typhimurium 14028s and *S*. *enterica* Senftenberg and compared it to the biofilm formation of *S*. *enterica* serovars Muenster and Brunei in an *in vitro* assay using 96-well microtiter plates. The biofilm formation of LT2 grown in LB was stronger than for LT2 grown in LM (**Figure 4**). The extent of biofilm formation of LT2 was comparable to *S. enterica* serovar Muenster, biofilm formation of *S.* Typhimurium 14028s was comparable to *S. enterica* serovar Brunei and weaker than biofilm formation of LT2 and *S.* Muenster (**Figure 4**). The LM seemed to affect the formation of biofilms in the case of LT2, *S.* Typhimurium strain 14028s, *S.* Muenster and *S.* Brunei. The only exception was *S*. Senftenberg, which formed remarkably extensive biofilms when grown in LM (**Figure 4**), although the biofilm formation in LB was comparable to that from LT2 or *S.* Muenster.

## Discussion

The ecology of *Salmonella* in soil is still largely unknown. In order to better understand the persistence of these bacteria in the environment, analysis of factors influencing their survival is essential. Microcosm experiments are good model systems to study the survival of *Salmonella* in soil under defined laboratory or greenhouse conditions. Diverse factors such as temperature, pH and humidity, have been demonstrated to influence the fate of *Salmonella* (Santamaría and Toranzos, 2003; Jacobsen and Bech, 2012). Unfortunately, experiments analyzing these factors, even if performed in similar experimental settings, often lead to contrary outcomes. One possible explanation might be the ways of preparing the inoculum for these experiments. We hypothesized that pregrowth conditions affect the survival of *Salmonella* in soil.

Since our long-term goal is to understand factors influencing the persistence of *Salmonella* in the phytosphere, in this report we developed and tested a new medium that contains lettuce material as the sole carbon source. In this medium, *Salmonella* does not encounter ideal growth conditions and cells are most probably stressed by antimicrobial compounds like phenolic metabolites or hydrogen peroxide released by plants (Barber et al., 2000; Wen et al., 2003; Toivonen et al., 2012; Barros and Saltveit, 2013; Ongeng et al., 2015). LM was used for cultivation of the inoculum to simulate preadaptation under laboratory conditions.

*Salmonella enterica* serovar Typhimurium strain LT2 was used as a model for *S. enterica* in our experiments. The influence of pregrowth in LM on the survival of LT2 in soil was analyzed in two independent microcosm experiments demonstrating significantly better survival when bacteria were introduced into soil after a pregrowth in LM compared to the respective bacteria pregrown in LB. These results indicated a preadaptation effect. Persistence in soil was monitored by CFU counts and results were confirmed in an independent qPCR approach in the first microcosm experiment. While cultivation-dependent quantification only detects cells able to form colonies, quantification of cells by culture-independent methods includes active cells, cells that died just recently and cells that entered the viable but non-culturable state in which they do not form colonies on routinely used solid nutrient media (Oliver, 2010). Therefore, numbers obtained by qPCR-quantification were about one order of magnitude higher than the CFU counts.

It was suggested that preadaptation to stress conditions increased the survival rate via the induction of traits related to stress-tolerance and that this mechanism helps *Salmonella* to cope with environmental stresses (Lee et al., 1995; Semenov et al., 2010; Shah et al., 2013). General resistance to stress depends on the transcriptional regulator RpoS and corresponding expression of down-stream genes. RpoS is accumulated in cells in the stationary phase or in cells suffering from starvation or other stresses (Battesti et al., 2011). *S.* Typhimurium LT2 is not able to elicit a sustained acid tolerance response (Lee et al., 1995) because the gene encoding RpoS starts with a rare TTG codon (Wilmes-Riesenberg et al., 1997), resulting in a low RpoS level. However, Shah et al. (2013) suggested that in *S.* Typhimurium LT2, the RpoS requirement for the induction of acid response could be circumvented by a preadaptation to cold stress. The authors showed that preadaptation of *S.* Typhimurium LT2 to cold stress leads to an induction of genes related to stress response and the repair pathways. Similar genes are induced in the RpoS-dependent responses to acid stress, for example, in *Escherichia coli* (Battesti et al., 2011). The principle that preadaptation to a given stress leads to increased survival upon other stresses, could be valid also for other *Salmonella* strains or other HP in general. However, since the detailed mechanisms are unknown, this phenomenon might depend on regulators other than RpoS (Shah et al., 2013). The effects of various stresses have also been analyzed with respect to the potential of human infections. Exposure to cold stress, for example, resulted in an increased adhesion and invasiveness of *S.* Typhimurium toward cultured intestinal epithelial (Caco-2) cells (Shah et al., 2014), and adaptation to mild acid stress protected against more acidic conditions as they are encountered in the human gut, and other stresses (Greenacre and Brocklehurst, 2006). Therefore, analyzing preadaption is not only important with respect to its effect on persistence in the environment, but also because it might constitute an enhanced risk for human infection.

In order to test whether an effect of LM-pregrowth on survival in soil could also be observed for other *Salmonella*, we included *S.* Typhimurium strain 14028s and another serovar *S.* Senftenberg in the microcosm experiment. Surprisingly, we could not observe an improved survival of these strains in soil. These results indicate a high strain specificity of LM-preadaptation. Strain- or serotype-specific behavior of *Salmonella* regarding, e.g., the survival in the agricultural or food production environment has been reported before. Andino et al. (2014) demonstrated strain dependent survival in poultry feed, and Fong and Wang (2016a) showed better survival of *S.* Hartford in peanut oil, peanut shell, and chia seeds compared to *S.* Typhimurium. Analyzing the persistence of *Salmonella* in whey protein powder, Farakos et al. (2014) found that *S*. Tennessee and *S*. Agona survived better than *S*. Montevideo, and that *S*. Typhimurium showed the lowest persistence. These differences between serovars should be taken into account when comparing results of studies that used different strains and pregrowth conditions to make predictions for risk assessments. Furthermore, the results obtained in this study for sandy soil might not be transferable to other soil types differing in their physicochemical and biological characteristics.

The mechanisms behind serovar- or strain-specific behavior with respect to preadaptation are so far unclear as the mechanisms that are involved in preadaptation are likely very complex and not yet understood. It is possible that the preadaptation effect observed in this study is due to altered physiology of preadapted cells. The *Salmonella* strains analyzed in this study were just sent for sequencing, as a comparison of their genomes might show differences that might contribute to the different behaviors of the strains observed in this study. Several studies indicated that biofilm formation enhances persistence of *Salmonella* in various environments (Vestby et al., 2009; Aviles et al., 2013; Patel et al., 2013; Yaron and Römling, 2014; Bridier et al., 2015). Therefore, we hypothesized that growth in LM affects LT2 in its biofilm formation ability. However, our results showed a more extensive biofilm formation of *S.* Typhimurium LT2 cells grown in LB, compared to cells grown in LM. Biofilm formation of LT2 in LM and LB was also compared to biofilm formation of *S.* Typhimurium 14028s and *S*. *enterica* serovars Senftenberg, Muenster, and Brunei. Among the strains and serovars tested in this study, only *S*. Senftenberg formed exceptionally extensive biofilms when grown in LM. The *S*. Senftenberg serovar used in this study was originally isolated from basil plants implicated in a produce-related outbreak in the UK (Pezzoli et al., 2007, 2008). Enhanced ability to form biofilms has been reported before for strains isolated from produce (Patel et al., 2013), as well as serovar- and strain-specific differences in attachment, for example, to plant leaves (Berger et al., 2009; Patel and Sharma, 2010; Yaron and Römling, 2014). The detailed mechanisms and factors responsible for the observed differences are so far unclear. The process of attachment to surfaces and biofilm formation is complex with a multitude of factors involved, and bacteria use several parallel mechanisms to allow attachment to a variety of surfaces under different conditions (Schikora et al., 2011; Yaron and Römling, 2014).

## Conclusion

This study demonstrated that pregrowth in the newly developed LM resulted in a better survival of *S.* Typhimurium LT2 in soil, in contrast to *S.* Typhimurium 14028s and *S.* Senftenberg. These results suggest that preadaptation could be an important factor determining the survival of *Salmonella* in the sandy soil that was analyzed here and should be considered in further experiments analyzing the fate of *Salmonella* in the environment. Interestingly, the LM differently influenced the biofilm formation of the *Salmonella* serovars tested. Particularly remarkable was the strongly increased ability of *S.* Senftenberg to form biofilms when grown in LM compared to LB. In contrast, the biofilm formation by *S*. Typhimurium LT2 was reduced in bioassays with growth in LM which might suggest that the improved survival of LT2 after LM-preadaption was likely not due to an improved biofilm formation.

This study demonstrated the limitations of results derived from experiments using laboratory model strains grown under ideal conditions, which are not representative for other strains as our data clearly showed that the medium used to pregrow *Salmonella* before inoculation into soil and to grow *Salmonella* during biofilm assays influenced both the ability to survive in soil as well as the ability to form biofilms in a strain-specific manner. We propose that this medium might be superior compared to other rich media typically used to grow enteric bacteria and thus can be recommended for studies investigating the ecology of enteric HP in soil and in the phyllosphere.

## Author Contributions

EF, JS, RG, AS, KS: designed the study; EF, JS: performed the experiments and analyzed the results; EF, SJ, AS, KS: wrote the manuscript.

## Conflict of Interest Statement

The authors declare that the research was conducted in the absence of any commercial or financial relationships that could be construed as a potential conflict of interest.
