# Supplementary material for: Persistence of Salmonella Typhimurium LT2 in Soil Enhanced after Growth in Lettuce Medium
Source: Front Microbiol. 2017 Apr 28;8:757. doi: 10.3389/fmicb.2017.00757 (PMC5408095; doi:10.3389/fmicb.2017.00757)
Supplement: Supplementary file 1 [file Data_Sheet_1.DOCX]

**Persistence of *Salmonella* Typhimurium LT2 in Soil Enhanced after Growth in Lettuce Medium**

Eva Fornefeld, Jasper Schierstaedt, Sven Jechalke, Rita Grosch, Adam Schikora and Kornelia Smalla*

*** Correspondence:** Kornelia Smalla: [kornelia.smalla@julius-kuehn.de](mailto:kornelia.smalla@julius-kuehn.de)

**Supplementary Data**


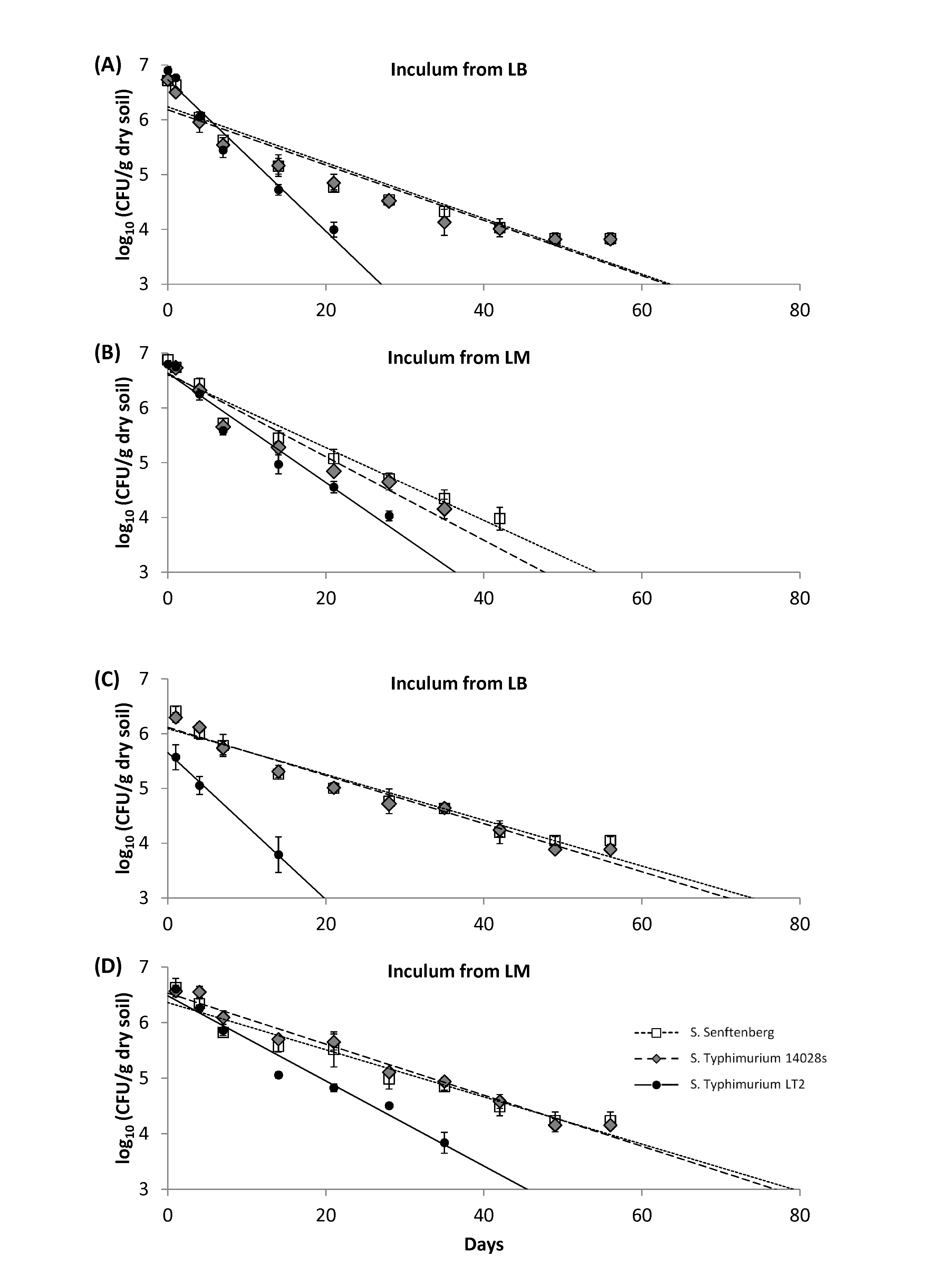


**Supplementary Figure S1:** CFU counts per gram dry DS soil of *Salmonella* inoculated from LB (A,C) and LM (B,D) in two independent repetitions of the experiment (first repetition: A, B; second repetition: C, D). Soil was sampled at 0, 4, 7, 10, 14, 21, 28, 35, 42, 49 and 56 days post inoculation (dpi). Lines correspond to linear regression models. The dotted lines represent the linear regression for *S.*Senftenberg without preadaptation from LB and with preadaptation from LM. Dashed lines represent the linear regression for *S.*Typhimurium 14028s without preadaptation from LB and with preadaptation from LM and solid lines represent the linear regression for *S.*Typhimurium LT2 without preadaptation from LB and with preadaptation from LM. The slopes of the linear regressions are significantly different (*p*<0.05) between LB and LM treatments only for *S.* Typhimurium LT2.

**Supplementary Table S2:** Slopes and correlation coefficients for linear regression models. Results were compared using Student’s *t*-test and differences considered significant when *p*-values were lower than 0.05.

|  |  |  | LM | | LB | |  |
| --- | --- | --- | --- | --- | --- | --- | --- |
|  |  | repetition | slope | R² | slope | R² | *p*-value |
| Microcosm experiment 1 | *S*. Typhimurium LT2 | plate counts  qPCR | -0.070  -0.077 | 0.98  0.94 | -0.089  -0.108 | 0.98  0.93 | 0.005  0.049 |
| Microcosm experiment 2 | *S*. Typhimurium LT2 | 1  2  3 | -0.079  -0.100  -0.077 | 0.97  0.96  0.96 | -0.102  -0.138  -0.134 | 0.93  0.97  0.996 | 0.009  0.002  N/A |
|  | *S*. Typhimurium 14028s | 1  2  3 | -0.063  -0.076  -0.046 | 0.96  0.92  0.98 | -0.062  -0.051  -0.044 | 0.94  0.91  0.96 | 0.152  0.152  0.378 |
|  | *S*. Senftenberg | 1  2  3 | -0.059  -0.066  -0.042 | 0.94  0.95  0.95 | -0.062  -0.051  -0.042 | 0.91  0.90  0.94 | 0.324  0.304  0.788 |
